# A highly elastic absorbable monofilament suture fabricated from poly(3-hydroxybutyrate-co-4-hydroxybutyrate)

**DOI:** 10.1038/s41598-023-30292-w

**Published:** 2023-02-25

**Authors:** Atsuhiko Murayama, Hidemasa Yoneda, Akira Maehara, Noriyuki Shiomi, Hitoshi Hirata

**Affiliations:** 1 Department of Human Enhancement & Hand Surgery, Nagoya University Graduate School of Medicine, 65 Tsurumai-cho, Shouwa-ku, Nagoya-shi, Aichi 466-8550 Japan; 2 Mitsubishi Gas Chemical Corporation Niigata Laboratory, Niigata, Japan; 3 Department of Personalized Medical Technology, Nagoya University Graduate School of Medicine, Nagoya, Japan

**Keywords:** Biomaterials, Therapeutics, Cardiovascular diseases, Inflammatory bowel disease

## Abstract

To address the growing demand for more elastic sutures free from unwanted knot loosening, we fabricated an absorbable monofilament suture from poly(3-hydroxybutyrate-co-4-hydroxybutyrate) and subjected it to physical property characterization and performance evaluation (in vitro and in vivo degradability tests and a porcine abdominal wall suture test). As this flexible, highly stretchable, and difficult-to-untie suture exhibited additional advantages of small knot size and medium to long-term bioabsorbability, it was concluded to be a safe alternative to existing monofilament sutures, with far-reaching potential applications.

## Introduction

Many medical devices used for soft tissue reconstruction have constant lengths, meaning that they are incapable of axial stretching. Therefore, they cannot be used for soft tissue reconstruction that requires the dynamic function of human joint tissue. For example, complications from their removal due to discomfort caused by poor distensibility, fistula formation, and neuroma are observed when inflexible artificial nerve conduits are used near finger joints^1–3^. Additionally, artificial ligaments contain fibers made of non-absorbent materials woven in a sheet or cylinder shape exhibiting sufficient linear tensile strength^4,5^. However, their ecological functions (extension and shape restoration) are not correctly executed. Therefore, the biomechanical reconstruction of joints using artificial ligaments is currently impossible. Ligament reconstruction grafting is primarily performed using autograft tendons, owing to their excellent rigidity and elasticity.

The elastic properties of sutures used in surgical procedures may prevent knot loosening and damage to tissue margins under shear^6,7^. Although conventional absorbable monofilament sutures possess smooth surfaces and are infection resistant, they tend to exhibit low knot security and their manipulation is sometimes challenging because they are more rigid and less flexible than multifilament sutures^8^. Many surgeons demand the development of absorbable monofilament sutures with easier handling and higher knot security.

Polyhydroxyalkanoates (PHAs) are biodegradable polymers of microbial origin. They exhibit high biocompatibility and eco-friendliness and are attractive in the biomedical engineering field as components of elastic medical devices^1^. Among currently available PHAs, only 4-hydroxybutyrate (4HB) polymers have clinical applications, mainly in sutures and cardiovascular stents^9^. Poly(3-hydroxybutyrate*-co-*4-hydroxybutyrate) (P(3HB*-co-*4HB)) becomes more extensible with an increase in the 4HB fraction^10^; therefore, it is a promising material for extensible medical devices. However, the development of P(3HB*-co-*4HB) has been hindered by the following factors: (i) the inability to chemically synthesize high-molecular-weight P(3HB*-co-*4HB)^11^, (ii) limited facilities available for conducting microbial synthesis, (iii) technical difficulty of culturing and purifying stable high-molecular-weight copolymers while maintaining a constant copolymerization ratio, (iv) challenges of scale-up bioprocessing, and (v) challenges of spinning the purified copolymers.

In this study, we solved the aforementioned problems and produced high-purity, high-molecular-weight copolymers. Furthermore, we developed elastic nonwoven fabrics and pouched structures that can be applied to fracture treatment^12^. Herein, the extensive characterization and suitability of highly elastic absorbable monofilament sutures fabricated from P(3HB-*co*-4HB) for practical applications are reported.

## Results

### Materials

**Figure 1:**
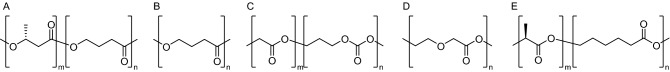
Chemical structures of the absorbable monofilament sutures used in this study: (**A**) P(3HB*-co-*4HB), (**B**) P(4HB), (**C**) PGA-TMC copolymer, (**D**) Poly*-p-*dioxanone, and (**E**) P(LA/CL).

The chemical structures of the repeating units of the absorbable monofilament sutures used in this study, P(3HB*-co-*4HB), Monomax® (P(4HB)), Maxon® (Poly(glycolide-co-trimethylene carbonate), PGA-TMC copolymer), PDS® II (Poly*-p-*dioxanone) and LACLON® (Poly(L-lactide/ε-caprolactone), P(LA/CL)) are presented in Fig. 1.

### Physical properties

**Figure 2:**
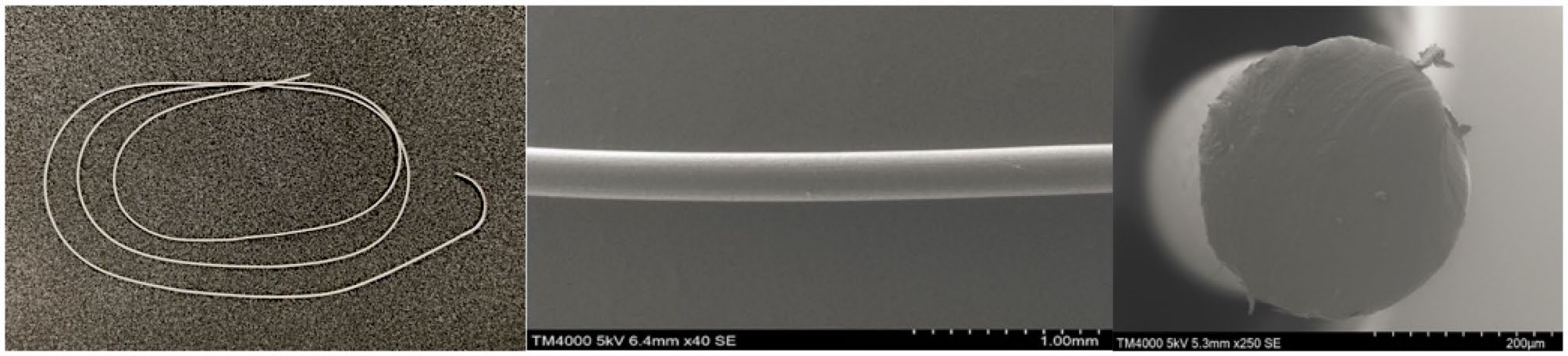
Photographic (left), side-view SEM (center), and cross-sectional SEM (right) images of the P(3HB*-co-*16 mol% 4HB) suture.

**Figure 3:**
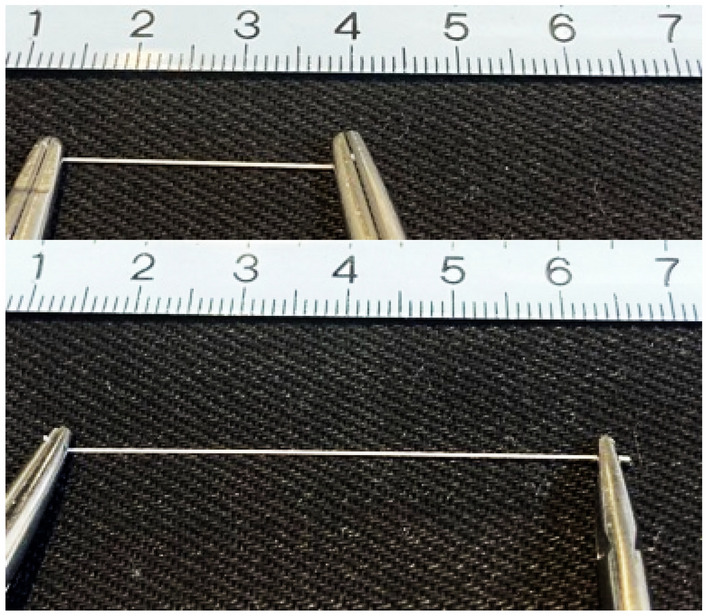
Photographs of the P(3HB*-co-*16 mol% 4HB) sutures without tension (top) and under tension (bottom).

**Figure 4:**
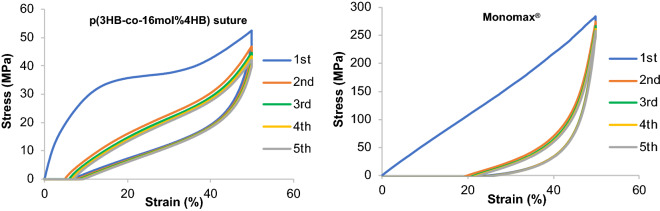
Tensile testing results of the P(3HB*-co-*16 mol% 4HB) (left) and Monomax® (right) monofilament sutures (n = 1).

The appearance and scanning electron microscopy images of the P(3HB*-co-*4HB) monofilament suture [United States Pharmacopoeia (USP) 2.5-0, 4HB content = 16 mol%] are shown in Fig. 2. Compared to Monomax® (USP 2-0), P(3HB*-co-*4HB) exhibited a 50% lower tensile strength (167 MPa), a ~ 50% lower Young’s modulus (261 MPa), and a two-fold higher breaking elongation (113%), meaning that it was supple and extensible (Table 1). Despite a substantial residual strain after 100% extension deformation (15%) and a plastic deformation exceeding its elastic limit, it did not break, unlike other sutures (Fig. 3). After 50% extension deformation in the cyclic test, the residual strains of Monomax® and the newly developed suture were 20 and 7%, respectively (i.e., the respective recoveries were 80 and 93%) (Fig. 4).

**Table 1:** Physical properties of absorbable monofilament sutures.

	Tensile strength (MPa)	Breaking elongation (%)	Young’s Modulus (MPa)	Residual strain after 50% extension deformation (%)	Residual strain after 100% extension deformation (%)
P(3HB*-co-*16 mol% 4HB) 2.5-0	167 ± 51	113 ± 22	261 ± 24	7*	15*
PDS® II 3-0	496 ± 20	59 ± 9	1563 ± 230	Rupture	Rupture
Monomax® 2-0	425 ± 132	88 ± 31	603 ± 56	20*	Rupture

Average ± 95% CI, * n = 1.

### Knot size and security

**Figure 5:**
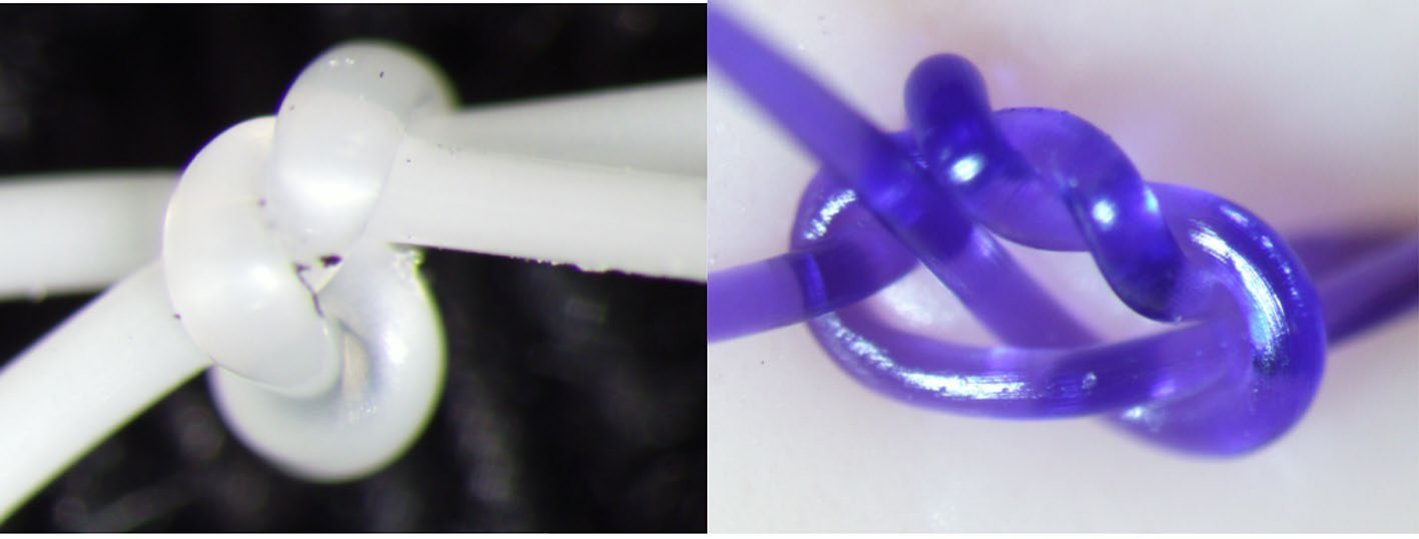
Photographs of surgical knots made using the P(3HB*-co-*4HB) (USP 2.5-0) (left) and PDS® II (USP 4-0) (right) absorbable monofilament sutures.

Using the P(3HB*-co-*4HB) monofilament suture (USP 2.5-0), a knot was formed and is shown in Fig. 5. Table 2 lists the related thread diameter and knot size. The corresponding data for PDS® II (USP 4-0) as a comparative example are presented in Fig. 5 and Table 3. Compared to PDS® II, the suture exhibits a shorter circumference (4.03 vs. 4.77 mm), a smaller knot area (0.843 vs. 1.20 mm^2^), and a smaller knot area/thread diameter ratio (3.28 vs. 7.32). However, it has a larger thread diameter (0.256 vs. 0.163 mm) and therefore a significantly smaller knot size (*P* = 0.0079).

**Table 2:** Knot sizes obtained for the P(3HB*-co-*16 mol% 4HB) suture.

Sample no.	Diameter (mm)	Perimeter^a^ (mm)	Area^b^ (mm^2^)	Area/Diameter ratio
A-1	0.268	4.99	1.06	3.96
A-2	0.261	4.06	1.00	3.82
A-3	0.250	3.77	0.726	2.90
A-4	0.244	3.52	0.694	2.84
A-5	0.258	3.79	0.737	2.86
Average	0.256	4.03	0.843	3.28

^a^Peripheral length of the knot outline as seen from above.

^b^Area enclosed by the knot outline as seen from above.

**Table 3:** Knot size of PDS® II.

Sample no.	Diameter (mm)	Perimeter^a^ (mm)	Area^b^ (mm^2^)	Area/Diameter ratio
B-1	0.165	4.96	1.32	8.02
B-2	0.161	4.24	0.917	5.69
B-3	0.165	5.30	1.35	8.16
B-4	0.163	5.10	1.40	8.58
B-5	0.162	4.24	0.993	6.14
Average	0.163	4.77	1.20	7.32

^a^Peripheral length of the knot outline as seen from above.

^b^Area enclosed by the knot outline as seen from above.

Table 4 lists the knot security factors (KSFs) of existing and newly developed absorbable monofilament sutures, revealing a lower KSF (2) for P(3HB*-co-*4HB), which is superior to those of currently used sutures.

**Table 4:** KSFs of absorbable monofilament sutures.

	USP	KSF
P(3HB*-co-*16 mol% 4HB)	2.5-0	2
PDS® II	2-0	3
	3-0	3
	4-0	3
LACLON® (P(LA/CL))	2-0	3
	3-0	3
	4-0	2

### In vitro degradability

**Figure 6:**
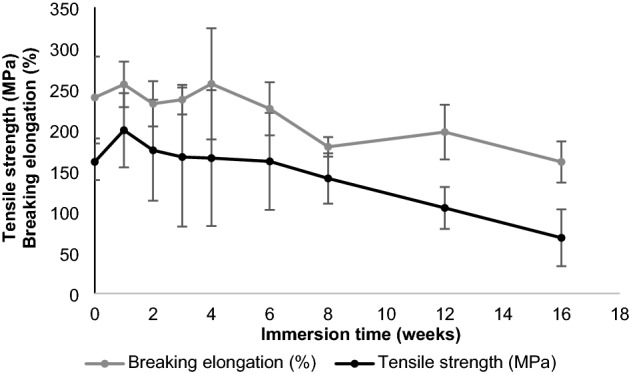
Retention of tensile strength and breaking elongation in vitro.

**Figure 7:**
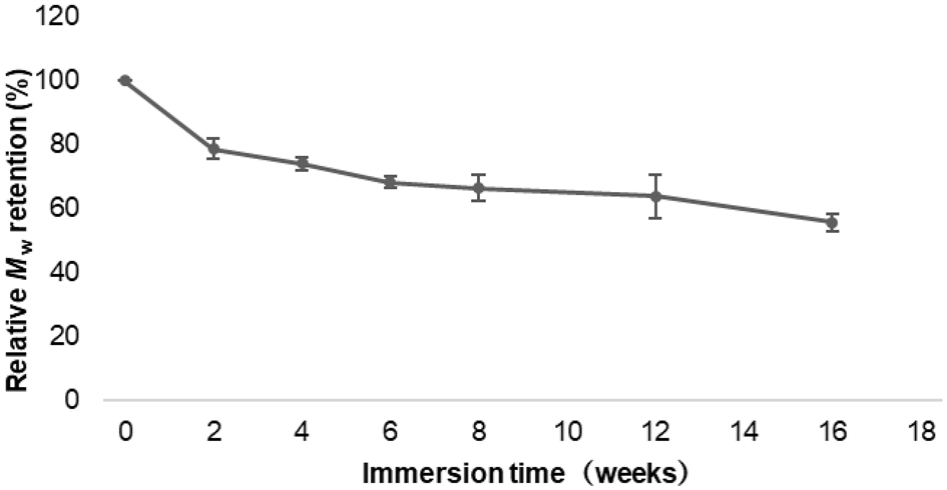
Retention of weight-average molecular weight in vitro.

A decrease in the initial linear tensile strength of the P(3HB*-co-*4HB) sutures from 161 to 104.6 MPa (i.e., by 35.0%) occurred after immersion in Dulbecco’s buffer for 12 weeks (Fig. 6). The fractional weight-average molecular weight (*M*_w_; value before immersion = 320,000, Da = 100%) as a function of immersion time (Fig. 7) exhibits a 50% decrease after 16 weeks. Furthermore, the P(3HB*-co-*4HB) suture maintained high elasticity and substantial elongation at break (160%, cf. initial value of 240%) (Fig. 6).

### In vivo degradability

**Figure 8:**
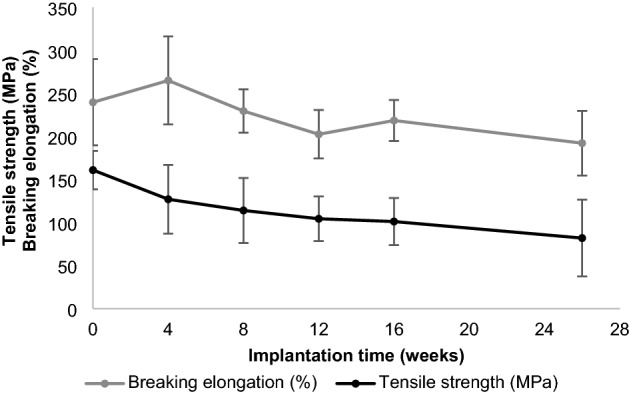
Retention of tensile strength and breaking elongation in vivo.

**Figure 9:**
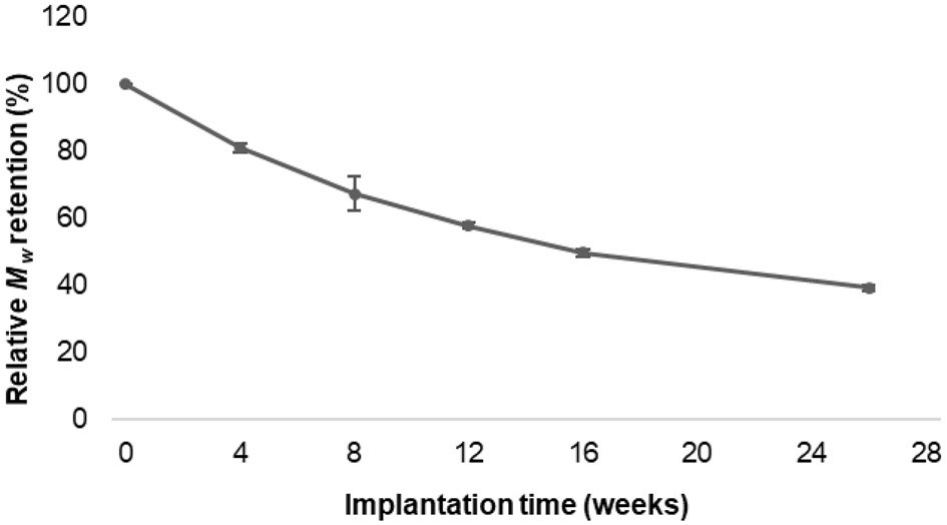
Retention of weight-average molecular weight in vivo.

The results of the tensile tests performed on P(3HB*-co-*4HB) sutures subcutaneously implanted in rats (residual strength ≈ 77% at week 4 and ≈ 63% at week 16) were consistent with those of the in vitro tests. The initial linear tensile strength was maintained at 50% (50% tensile strength holding period) for 26 weeks (Fig. 8). The fractional decrease in *M*_w_ with increasing implantation time (Fig. 9) shows a 50% decrease, 16 weeks after implantation. Furthermore, the elongation at the break of the P(3HB*-co-*4HB) suture remained close to 200%, 26 weeks after implantation (Fig. 8).

### Performance in pig abdominal wall suture test

**Figure 10:**
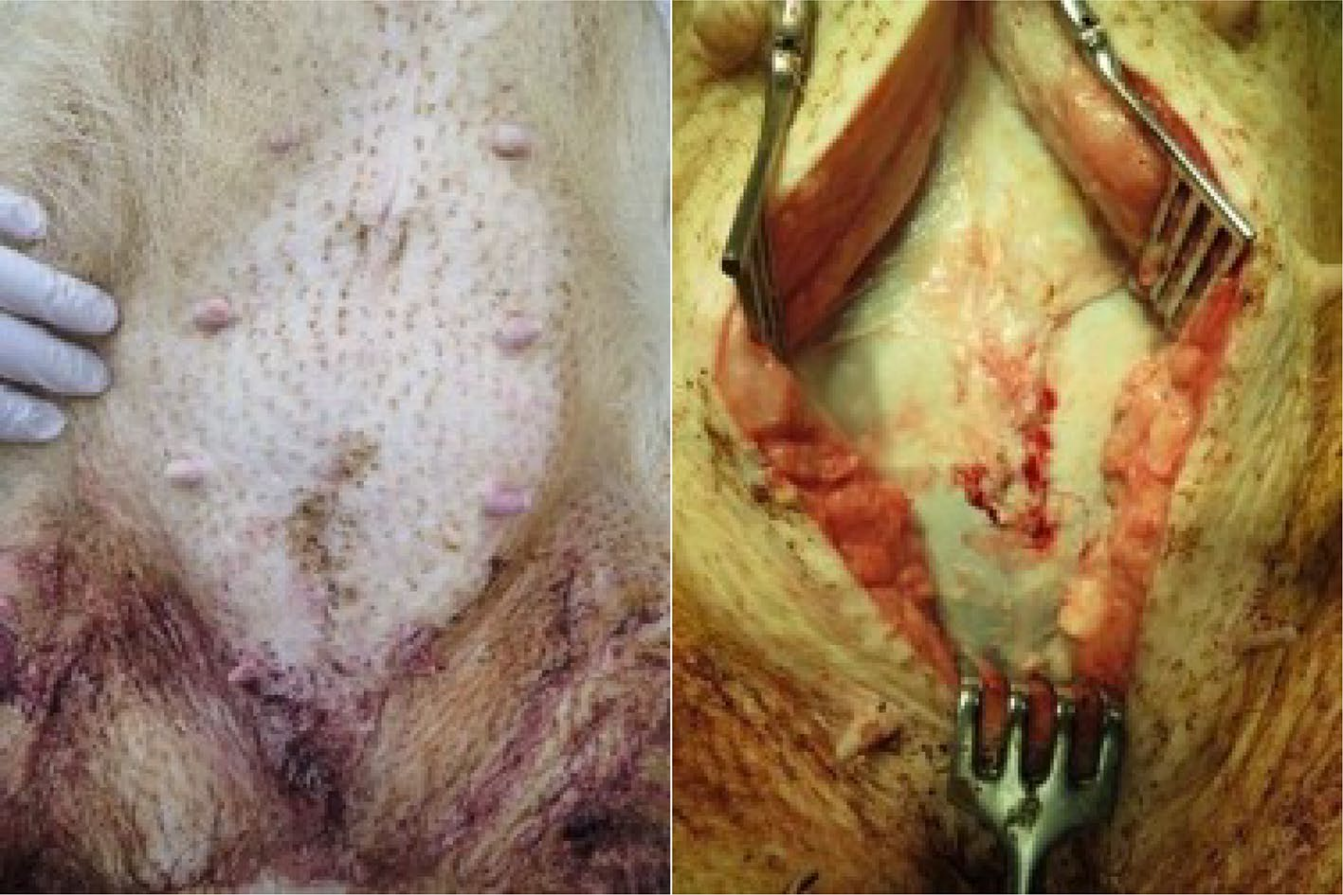
Abdominal (left) and abdominal wall (right) images of Microminipigs® seven weeks after surgery.

**Figure 11:**
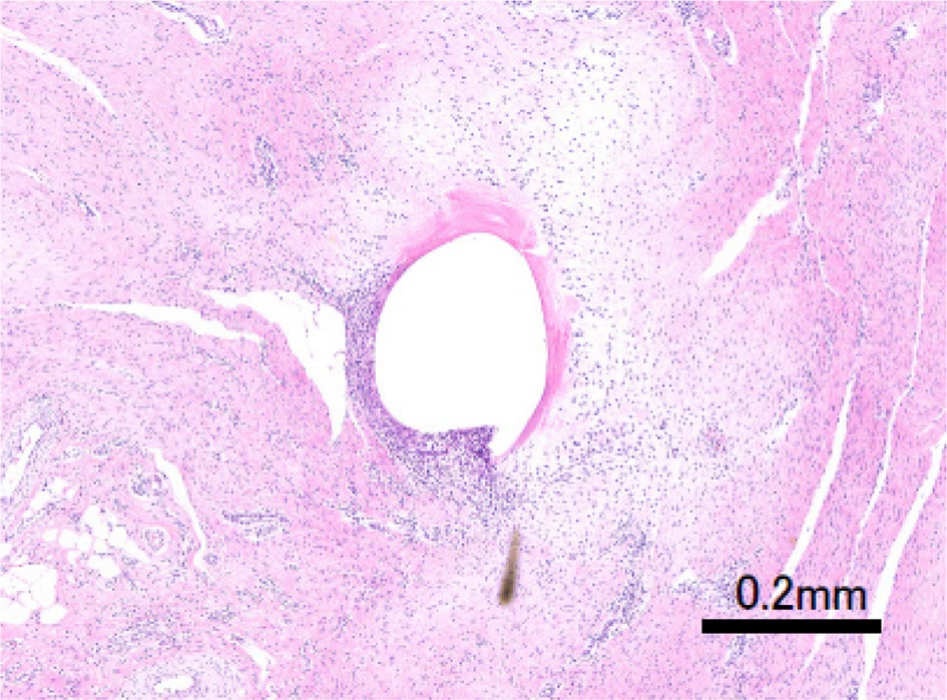
Microscopic image of HE-stained P(3HB*-co-*4HB) perisuture tissue. The suture (white oval) is surrounded by purple-stained inflammatory cells. The accumulation of inflammatory cells around the new suture was less pronounced than that around the Maxon® and Monomax® sutures.

**Figure 12:**
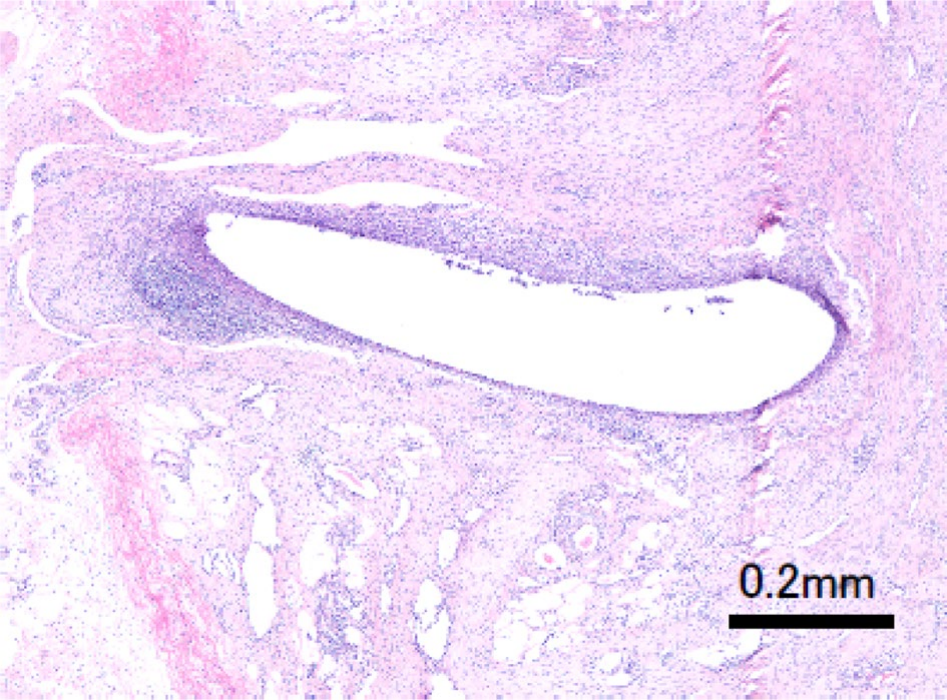
Microscopic image of HE-stained Maxon® perisuture tissue.

**Figure 13:**
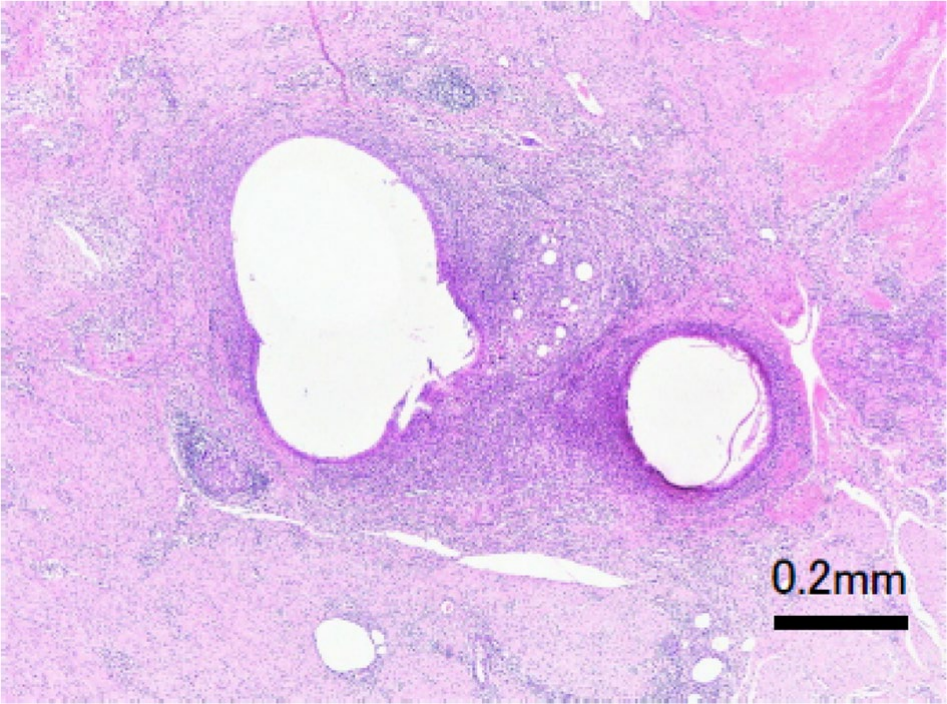
Microscopic image of HE-stained Monomax® perisuture tissue.

Seven weeks after integrating the abdominal wall suture, no substantial complications (signs of infection, wound dehiscence, abdominal wall incisional hernia, or adhesions) were macroscopically observed at any of the three suture sites (Fig. 10). The histological evaluation of the P(3HB*-co-*4HB) suture by an independent pathologist returned a score of unity for inflammation, necrosis, and fibrous thickening (Table 5). The histological images of the weak expansion of the hematoxylin and eosin (HE) staining of the surrounding tissue of the P(3HB*-co-*4HB), PGA-TMC copolymer, and P(4HB) sutures are shown in Figs. 11, 12, and 13, respectively. These results suggest that after seven weeks, the P(3HB*-co-*4HB) suture exhibited less inflammation than the other two absorbable monofilament sutures and was also non-inferior in terms of necrosis and fibrous thickening.

**Table 5:** Histological evaluation of the region around the porcine abdominal wall suture seven weeks after surgery.

	P(3HB*-co-*4HB)	PGA-TMC copolymer	P(4HB)
Inflammation	1	2	3
Necrosis	1	1	1
Fibrous thickening	1	1	2

## Discussion

Compared to its commercially available analogs, the P(3HB*-co-*4HB) monofilament suture had a lower Young's modulus, better extensibility (almost twice the breaking elongation), and a smaller knot size (i.e., a higher loosening resistance). Additionally, the newly developed suture did not break when extended to twice its original length, exhibiting an 85% length restoration upon relaxation. As its 50% tensile strength retention period determined by the in vivo test was close to 26 weeks, the suture was concluded to be of a gradually hydrolyzed medium to long-term absorbable type. Compared to existing absorbable sutures, it did not cause infection or incisional hernias in the porcine abdominal wall test and was histologically non-inferior in inducing inflammation.

The Young’s moduli of existing absorbable monofilament sutures substantially exceeded those previously reported for visceral organs (stomach, large intestine, and bladder), subcutaneous and other tissues (≤ 10 MPa), and tendons (450 MPa)^13–15^. The use of these sutures results in tissue damage due to stiffness mismatch; mechanical failure and cheese wiring (tendon cut-through) were observed after repairing the rotator cuff tear^16–18^. Moreover, cheese wiring causes postoperative rerupture after repairing the rotator cuff and meniscus^19,20^. A long-term evaluation of open meniscus repair over 10 years showed that 21–29% of retears occurred postoperatively^21–23^, while Beamer et al. reported tissue failure due to suture stiffness^24^. Reducing the stiffness mismatch can therefore decrease the possibility of rupture after tissue repair, with the newly developed suture well-suited for this purpose.

The P(3HB*-co-*4HB) suture gained more elasticity by increasing the 4HB fraction. Previously, the breaking elongation of a copolymer film containing 16 mol% 4HB was 444%^10^, while copolymer films containing ≥ 30 mol% 4HB exhibited rubber-like elastic behavior^25^. Given its advantageous physical properties, the P(3HB*-co-*16 mol% 4HB) monofilament suture developed herein could accommodate temporary tissue swelling and deformation due to body motion, and also extend without overtightening which prevents a decrease in blood flow to the tissue.

Moreover, unlike conventional absorbable monofilament sutures, the newly developed suture did not suffer from looseness. Conventional suture knots loosen because of impliability and incompliance, while the number of ligations or the ligature force can be increased to prevent this. Sufficient knot security is achieved using four or more ligations^26^, although here, the large knot size and the negative influence on the surrounding tissue pose concerns. Van Rijssel et al. semiquantitatively evaluated the perisuture tissue response in rats. They reported that the knot size increased 4- to 6-fold with an increase of two suture sizes, which triggered an increased tissue response around the suture^27^. Herein, the small knot size of the suture was expected to reduce the reaction of the surrounding tissue. KSFs of 2–4 are considered excellent for monofilament sutures^28^. The KSF value of our suture (2) indicated higher knot security than that achieved by PDS® II and LACLON®. The high knot security and the decreased knot number of our developed suture may achieve shorter operative times, e.g., for endoscopic suturing in intraperitoneal surgery, which is a basic yet technically demanding procedure^29^.

The 50% tensile strength-duration of synthetic absorbable monofilament sutures in animals has been reported as ~ 6 weeks for PDS® II (3-0) and 12–16 weeks for Monomax® (3-0)^28^. The P(3HB*-co-*16 mol% 4HB) monofilament suture (2.5-0) reached this at ~ 26 weeks and was, therefore, more durable and more suitable for suturing slow-healing wounds than existing synthetic absorbable monofilament analogs. From the chemical structure of P(3HB-*co*-4HB) shown in Fig. 1, it is apparent that it is a relatively hydrophobic material compared with the aforementioned commercial monofilament sutures. Although foreign body reactions have been reported for many absorbable sutures^30^, in the porcine suture model the abdominal wall healed without rupture, seven weeks after surgery. For various P(3HB*-co-*4HB) materials, tests on cultured cells and implantation tests on experimental animals indicated no obvious toxicity^31^. Both 3HB and 4HB, which are the degradation products of P(3HB*-co-*4HB), existed in vivo^32–34^. As the degree of inflammation around the new suture was histologically small and the suture was slowly hydrolyzed in vivo, it was concluded that interference with tissue healing did not occur.

This study has several limitations. In the porcine abdominal wall suture test, the histological evaluation could only be performed seven weeks after the operation. Therefore, it is unclear when maximum inflammation due to the foreign body reaction of the new suture occurred, and whether the biocompatibility of our suture is superior to that of existing absorbable sutures throughout the postoperative period. The results of in vivo and in vitro degradation studies do not allow an estimation of the time required for complete bioabsorption of P(3HB*-co-*16 mol% 4HB), while the influence of pH on this degradation is also unclear. Additionally, regarding knot size comparisons, a two-dimensional assessment was performed using images captured directly above the knots.

The absorbable P(3HB*-co-*16 mol% 4HB) monofilament suture is characterized by high flexibility and elasticity, small knots that are difficult to untie, and high biocompatibility and safety. These features are useful when suturing fragile tissues or when performing laparoscopic internal ligation. Thus, the newly developed product complements the lack of extensibility observed for conventional absorbable monofilament sutures and may significantly change the applications of absorbable monofilament sutures.

## Methods

### Materials

**Figure 14:**
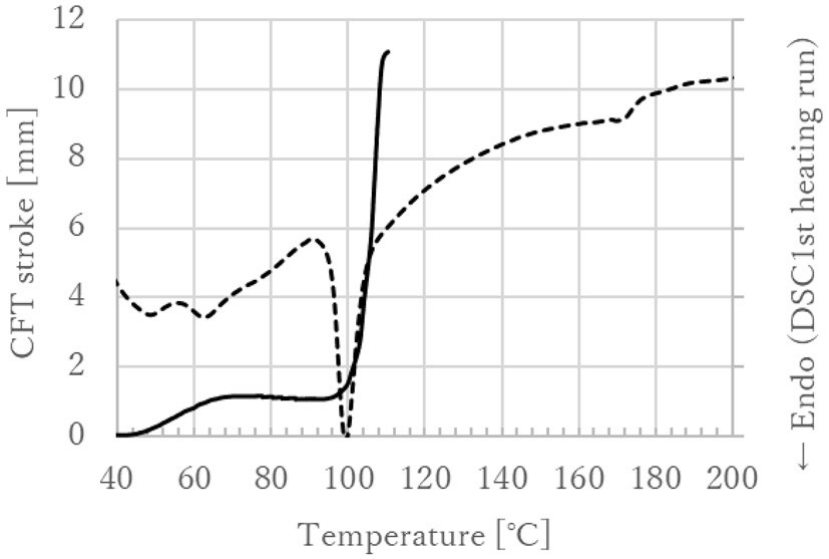
Flow curve (solid line) and DSC curve (dashed line) of P(3HB*-co-*16 mol% 4HB) using the flow tester heating method. The melting point (T_m_) is ~ 60–175 °C. The glass-transition temperature (T_g_) is below 0 °C and cannot be observed in the first heating of DSC.

High-molecular-weight P(3HB*-co-*16 mol% 4HB) (*M*_w_ ≈ 600,000 Da; Mitsubishi Gas Chemical Corporation) was obtained using a combination of fermentation (biosynthesis) and purification methods and then processed into fibers using partial melt-spinning^35,36^. This method produces polymer molded products and involves polymers that contain lamella crystals with different lamella thicknesses. During this method, a melt molding procedure takes place in a temperature range where some lamella crystals are melted and fluidized, whereas the rest of the lamella crystals remain unchanged^36^. Figure 14 shows a flow curve and a differential scanning calorimetry (DSC) curve of P(3HB*-co-*16 mol% 4HB) using the flow tester heating method^36^. The chemical compositions (mol% 4HB) of the copolymers used in this study were determined by the gas chromatography method described in the international patent WO 2019/044836A1^37^. The standard was calibrated using 3HB methyl and gamma-butyrolactone.

All animal experiments complied with the basic ethical guidelines of the Center for Animal Research and Education and were approved by the Institutional Animal Care and Use Committee of Nagoya University. Moreover, we certify compliance with the ARRIVE guidelines.

### Physical properties

The tensile strength, breaking elongation, Young’s modulus, and residual strain after 100% deformation of the P(3HB*-co-*16 mol% 4HB) monofilament sutures (USP 2.5-0), PDS® II (USP 3-0), and Monomax® (USP 2-0) were measured using a tensile testing machine (AGS-50 NX, Shimadzu Corporation, Kyoto, Japan). In the corresponding tests (*n* = 3), 120-mm-long fibers were fixed using a length of 10 mm above and below, and an assessment was performed at a 100-mm-distance between the chucks of the tensile testing machine using a tensile speed of 10 mm min^−1^. The residual strain (*S*_100_, %) was calculated from the tensile elongation recovery (*R*_100_, %) as *S*_100_ = 100% − *R*_100_. After the suture was stretched to a 100% strain (200 mm, which corresponds to two times the initial length or a 100 mm displacement length), it was contracted by moving the gripper at a constant speed until the pre-stretch length was reached. Assuming that the displacement length at the first time point of the second stretch (which is assumed to approximately equal the end point of the first stretch) is *X*_100_ mm, the tensile elongation recovery is given by *R*_100_ (%) = 100% × [200 − (*X*_100_ + 100)]/100.

In the cycle test (n = 1), both ends (10 mm) of the 120-mm-long suture were gripped, and the suture was stretched to a 50% strain at an initial length (chuck-to-chuck distance) of 100 mm at room temperature (23 °C) using a tensile speed of 20 mm min^−1^. The gripper was then moved to the original length at the same speed to shrink the suture. This procedure was repeated five times. Error analysis was performed with a confidence interval of 95% (95% CI).

### In vitro degradation test

Each of the eight ethylene oxide gas (EOG)-sterilized P(3HB*-co-*4HB) sutures with a thread diameter of 0.200–0.249 mm and a length of 300 mm was immersed in Dulbecco’s phosphate buffered saline (pH 7.4, 37 °C) contained in a conical tube. After immersion for 1, 2, 3, 4, 6, 8, 12, and 16 weeks, the sutures were removed and vacuum-dried. The 300-mm-long suture was cut at 30-mm intervals. Both ends of the suture were grasped and tensile tests (tensile strength and breaking elongation evaluations) were performed at room temperature (23 °C), under an initial length (chuck-to-chuck distance) of 10 mm, and a tensile speed of 10 mm min^−1^ (*n* = 3–10), until the suture ruptured. Molecular weight measurements were performed on sutures at 2, 4, 6, 8, 12, and 16 weeks after immersion. Week 0 (initial value) was defined as no immersion in the buffer solution. The molecular weights were determined by gel permeation chromatography using an HPLC Prominence system (Shimadzu Corporation, Kyoto City, Japan). The tensile properties were determined using an AGS-50 NX machine. The error analysis was performed with a 95% CI.

### In vivo degradation test

Fifteen rats (Male F 344/NSLc, 20 weeks old) were prepared, the dorsal skin was incised 80 mm along the spinal column, and EOG-sterilized P(3HB*-co-*4HB) sutures (diameter = 0.200–0.249 mm, length = 100 mm) were implanted into the subcutaneous tissue. At 4, 8, 12, 16, and 26 weeks after implantation (three animals per time point), the sutures were removed and vacuum-dried for tensile testing and molecular weight determination (*n* = 9–12). The initial value (0 weeks) was defined as no implantation. Tensile tests and molecular weight measurements were performed using the same conditions and equipment as those employed in the in vitro tests. Similarly, the error analysis was performed with a 95% CI.

### Evaluation of knot size

Five P(3HB*-co-*4HB) sutures (USP 2.5-0) and five PDS® II sutures (USP 4-0) were prepared, and their diameters were measured using a dial thickness gauge (Techlock Co., Ltd., SM-1201 L Model, scale interval = 0.001 mm). Measurements were performed in three locations (1/4, 1/2, and 3/4 of the total length), and the average was defined as the thread diameter. Surgical knots were prepared on an artificial skin sheet made of a soft elastomer and tightened using a force of 5 N (Standard Model Digital Force Gauge, ZTS-100 N, IMADA Co., Ltd.). Each knot (five samples in total) was photographed from above using a camera (DP 26, Olympus Co., Ltd.) attached to a stereomicroscope (SZX7, Olympus Co., Ltd.). The knot size (knot perimeter and area) was determined using an image analysis software (cellSens, Olympus Co., Ltd.) (Fig. 4) and expressed as the knot area and the knot area/thread diameter ratio. The knot area/thread diameter ratio was processed using the statistical software EZR version 4.0.4 (Saitama Medical Center, Jichi Medical University, Saitama City, Japan). The lack of normality in both groups was tested using the Shapiro–Wilk test. The Mann–Whitney test was then performed between the two groups, and *p* values of < 0.05 indicated significant differences.

### Evaluation of knot security

KSF was used as an index of knot unraveling difficulty^28,38^. P(3HB*-co-*16 mol% 4HB) (2.5-0), PDS® II (2-0, 3-0, 4-0), and LACLON® (2-0, 3-0, 4-0) sutures were wound around a 2.9-cm-diameter plastic tube, tightly tied with a surgical knot, and cut at the side opposite the knot to create a single thread. Both sides were attached to a tensile tester and pulled at a rate of 100 mm min^−1^. Multiple sets of 10 samples were prepared, and if one of the 10 samples had a knot untied, a single nodule was added above the surgical knot until no knot could be untied. KSF was defined as the number of single nodules added until all 10 knots could not be untied, i.e., denotes the number of knots required to maintain adequate ligation. No statistical processing was performed in this experiment.

### Pig abdominal wall suture test

Pregnant 32-week-old female Microminipigs® (Fuji Micra Co., Ltd., Fujinomiya City, Japan) were used for abdominal wall suturing with P(3HB*-co-*4HB) sutures and two existing absorbable monofilament sutures. Macroscopic and microscopic assessment of any occurring complications and inflammatory reactions followed. After delivering the fetus by cesarean section, a 12-cm longitudinally incised abdominal wall was sutured. Three apical P(3HB*-co-*4HB) sutures, two central PGA-TMC copolymer sutures, and three caudal P(4HB) sutures were used. The presence or absence of complications (signs of infection, wound dehiscence, abdominal wall incisional hernia, and adhesions) at the suture site was macroscopically observed seven weeks after suturing. Additionally, the abdominal wall (including the suture part) was collected, various suture parts were cut, and after each paraffin fixation, HE staining was carried out by the standard procedure. The extent of inflammation was assessed using a light microscope. The pathologist blindly evaluated the tissues in terms of inflammation, necrosis, and fibrous thickening on three different, nonadjacent sections using a scale of 0–4 (0: No inflammatory response, no necrosis, no thickening; 1: Very small inflammatory response, very little necrosis, very little thickening; 2: Inflammatory response, necrosis, thickening; 3: Strong inflammatory response, strong necrosis, strong thickening). The samples were reassessed two weeks after the first observation, and if the results of the first assessment were different from those of the second one, a third assessment was performed two weeks later, for final determination. No statistical processing was performed in this test.

## Data Availability

The data supporting the findings of this study are available on request from the corresponding author but are not publicly available due to a joint research and development agreement.
